# Robust gesture recognition based on attention-deep fast convolutional neural network and surface electromyographic signals

**DOI:** 10.3389/fnins.2024.1306047

**Published:** 2024-07-10

**Authors:** Chuang Lin, Yuhao Wang, Ming Dai

**Affiliations:** ^1^School of Information Science and Technology, Dalian Maritime University, Dalian, China; ^2^School of Artificial Intelligence, Shenzhen Polytechnic University, Shenzhen, China

**Keywords:** electrode shift and damage, sEMG, gesture recognition, myocontrol, deep learning

## Abstract

The surface electromyographic (sEMG) signals reflect human motor intention and can be utilized for human-machine interfaces (HMI). Comparing to the sparse multi-channel (SMC) electrodes, the high-density (HD) electrodes have a large number of electrodes and compact space between electrodes, which can achieve more sEMG information and have the potential to achieve higher performance in myocontrol. However, when the HD electrodes grid shift or damage, it will affect gesture recognition and reduce recognition accuracy. To minimize the impact resulting from the electrodes shift and damage, we proposed an attention deep fast convolutional neural network (attention-DFCNN) model by utilizing the temporary and spatial characteristics of high-density surface electromyography (HD-sEMG) signals. Contrary to the previous methods, which are mostly base on sEMG temporal features, the attention-DFCNN model can improve the robustness and stability by combining the spatial and temporary features. The performance of the proposed model was compared with other classical method and deep learning methods. We used the dataset provided by The University Medical Center Göttingen. Seven able-bodied subjects and one amputee involved in this work. Each subject executed nine gestures under the electrodes shift (10 mm) and damage (6 channels). As for the electrodes shift 10 mm in four directions (inwards; onwards; upwards; downwards) on seven able-bodied subjects, without any pre-training, the average accuracy of attention-DFCNN (0.942 ± 0.04) is significantly higher than LSDA (0.910 ± 0.04, *p* < 0.01), CNN (0.920 ± 0.05, *p* < 0.01), TCN (0.840 ± 0.07, *p* < 0.01), LSTM (0.864 ± 0.08, *p* < 0.01), attention-BiLSTM (0.852 ± 0.07, *p* < 0.01), Transformer (0.903 ± 0.07, *p* < 0.01) and Swin-Transformer (0.908 ± 0.09, *p* < 0.01). The proposed attention-DFCNN algorithm and the way of combining the spatial and temporary features of sEMG signals can significantly improve the recognition rate when the HD electrodes grid shift or damage during wear.

## 1 Introduction

The interaction between humans and machines has gradually gained attention as a result of the development of robots and human-machine collaboration (Küçüktabak et al., 2021). Electromyography (EMG) signals are a convenient and reliable way to establish a link between external machines and human motor intention (Artemiadis and Kyriakopoulos, 2010). They can be used to control individual devices or prostheses (Englehart and Hudgins, 2003). Surface electromyography (sEMG) signals are obtained by quantitatively measuring muscle activity, typically collected non-invasively through electrodes, providing a simple method for human-machine interaction. Nowadays, human-machine interaction systems based on surface electromyography (sEMG) signals have been used in many fields such as prosthetic control, exoskeletons, and industrial robots. Meanwhile, sEMG gesture recognition has become the core of muscle connective interface (MCI) (Zheng et al., 2022).

Surface electromyography (sEMG) signals are generally categorized as sparse multi-channel (SMC) or high-density (HD) based on the number of sensors for measuring. The SMC method is more sensitive to the placement position of the electrodes due to the small number of electrode channels (Amma et al., 2015). Incorrect placement positions may affect the acquisition of sEMG signals, it limits the application of sEMG in muscle connective interface (MCI). In contrast, the HD method has numerous and dense electrode arrangements which can cover a larger range (Amma et al., 2015; Stango et al., 2015). Researchers have proposed various prosthetic control algorithms over the past many years (Fougner et al., 2012; Xu and Xiong, 2021). Nowadays, deep learning is being used in the processing of sEMG signals as artificial intelligence technology advances. Compared to traditional recognition methods, deep learning-based recognition methods have greater advantages due to their good expansibility and accuracy, as well as their end-to-end capabilities. As a type of time series signal, sEMG signal often leads to a lack of spatial information when used for gesture recognition. Geng et al. (2016) introduced the concept of sEMG images composed of HD-sEMG spaces to utilize spatial information, resulting in improved recognition results. In practical applications, high-density electrodes often experience electrode shift and damage, resulting in a decrease in the accuracy of gesture recognition. We should consider researching new methods to avoid this situation.

Tkach et al. (2010) studied the effects of different interference methods on electromyography signals, the results showed that moving electrode positions would reduce the classification accuracy of most features. Young et al. (2012) proposed that increasing the distance between electrodes from 2 cm to 4 cm to improve pattern recognition accuracy. In recent years, researchers have attempted to achieve gesture recognition based on neural networks. Wei et al. (2019) used multi-view deep learning method, achieving sparse multichannel sEMG gesture recognition. Zhang et al. (2019) improved the performance of gesture recognition when electrode position is moved or damaged by utilizing featured sEMG image and CNN. Ameri et al. (2020) proposed a supervised adaptation method based on convolutional neural networks (CNNs) and transfer learning to solve the problem of insufficient calibration data due to short training time of classification and regression based control schemes. Kim et al. (2021) studied the effects of posture groups, feature vectors, and electrode shift on hand posture recognition, and found that the accuracy of recognition increased as the number of electrode shift training increased. Wu et al. (2022) proposed a new method that can retain critical and stable information when the electrodes shift. Wang et al. (2023) enhanced the flexibility of the muscle interface through one-shot learning. By using HD-sEMG images for EMG pattern recognition, enabling easy switching between different usage situations and reducing the burden of retraining.

We propose a new model, which we named attention-deep fast convolutional neural network (attention-DFCNN). The model can resist the impact of electrode displacement by utilizing the temporal and spatial characteristics of HD-sEMG signals. The HD-sEMG signals are transformed into featured sEMG images and then put into the attention-DFCNN model. Compared to traditional methods and classical deep learning methods, attention-DFCNN exhibits better robustness and higher accuracy in gesture recognition against electrode shift and damage.

## 2 Methods

### 2.1 Dataset and preprocessing

#### 2.1.1 Dataset

**Figure 1:**
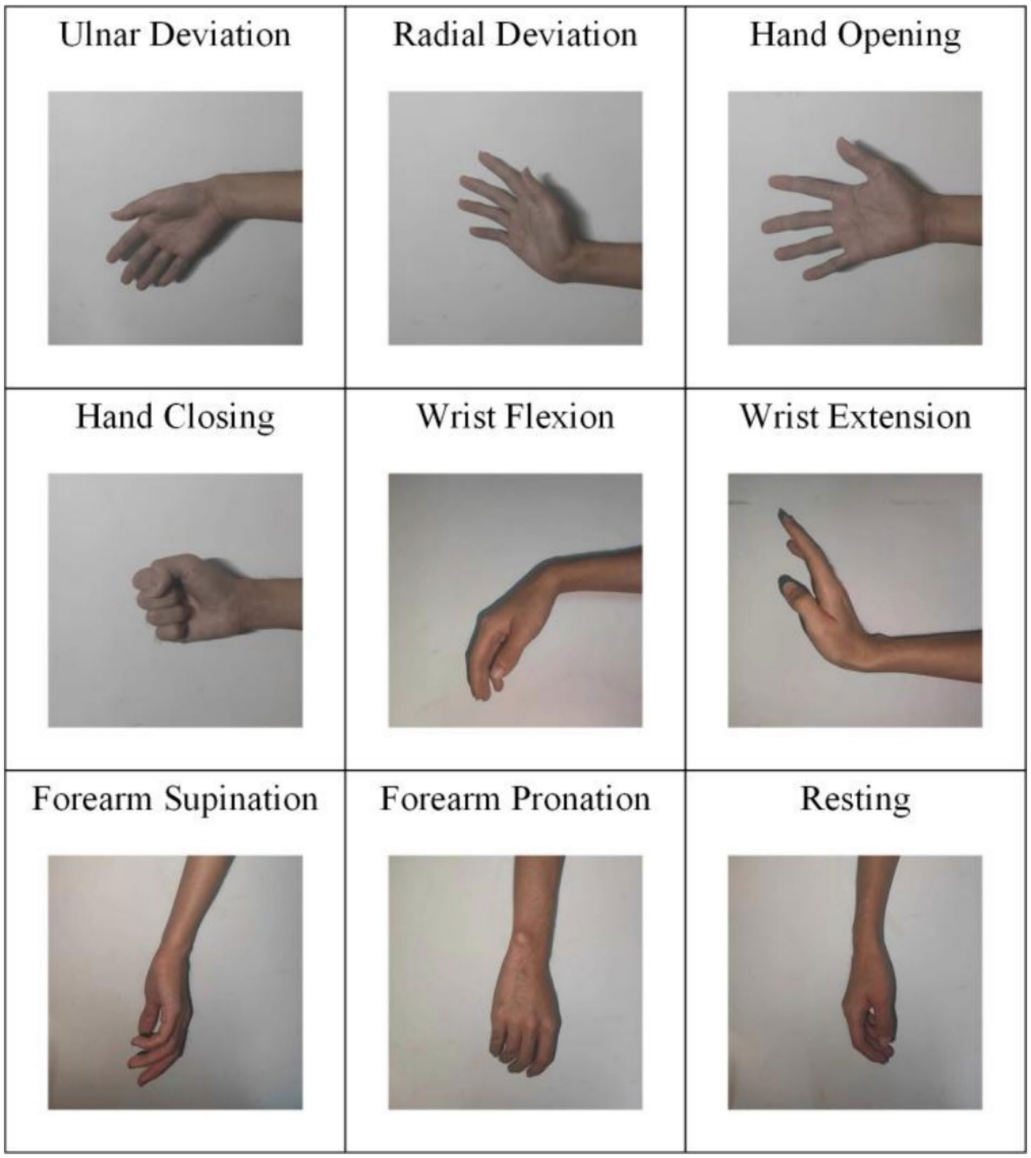
The photo display of nine gestures.

The University Medical Center Göttingen contributed the data used in the experiment (Stango et al., 2015). They were collected from three male able-bodied subjects and four female able-bodied subjects, and one amputee with unilateral trans-radial traumatic amputation. The average age of the able-bodied subjects was 29 years old, and the age of the amputee was 78 years old (53 years after the amputation). The sEMG signals were collected by using OT Bioelettronica with 192 electrodes. The electrode was placed at one-third of the forearm near the elbow joint. The signals were amplified with a gain of 500 and sampled at 2,048 Hz. XSENS Technologies was placed on the subjects to track their motion during the experiment. It can record the center time of each trial. The reference electrode was positioned on the wrist, and the HD-sEMG grid was positioned on the forearm. In the experiment, nine gestures (see Figure 1) were classified, including ulnar deviation, radial deviation, hand opening, hand closing, wrist flexion, wrist extension, forearm supination, forearm pronation and the resting. Four repetitions were recorded for each gesture, and each subject performed the experiment 4 times. Every subject received a brief training on the movements demonstrated on a computer screen. For the transradial amputee, the number of electrodes was reduced by two rows of the grid due to the short stump, so that the electrodes comprised 6 rows and 24 columns (144 electrodes). The reference electrode was placed on the elbow. The motion trackers were placed on the contralateral arm. The subject performed mirror movements, attempting to replicate the tasks of the able-limb with the phantom limb. For more details of the data, please refer to the article (Stango et al., 2015).

#### 2.1.2 Preprocessing

Because the tracking system recorded the sample corresponding to the central time of each movement, a static window of 2.2 s (4,500 samples) centered on that sample was selected as the task window. A 150 sample window was utilized to compute the root mean square (RMS) values of the sEMG. So, a task window (containing 4,500 samples) is evenly divided into 30 parts, and a total of 30 sets of RMS values were calculated. The RMS is defined as:


$$RMS=\sqrt{\frac{\sum_{i=1}^{n}x_{i}^{2}}{n}}$$

In order to conduct research on electrodes shift and electrodes damage, we simulated two scenarios using collected datasets: electrodes shift 10 mm and electrodes shift 10 mm with damage in 6 channels.

**Figure 2:**
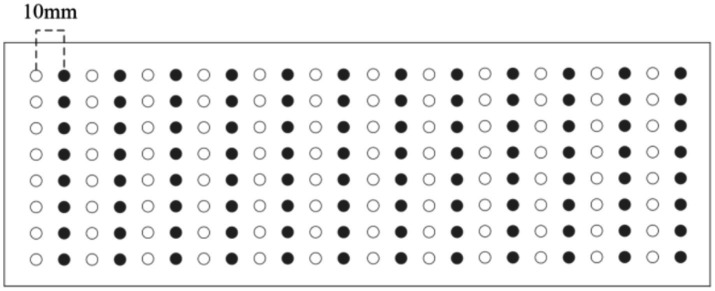
Inwards/onwards shift 10 mm.

**Figure 3:**
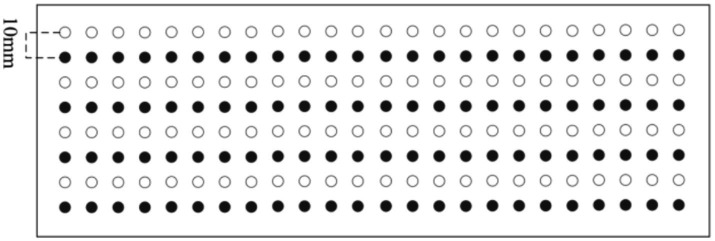
Upwards/downwards shift 10 mm.

To simulate electrodes shifting 10 mm, we divide the datasets into training and testing sets in an interlaced manner based on their spatial arrangement. In the shift inwards, the black dots represent training electrodes, while the white dots represent testing electrodes. In the shift onwards, the white dots correspond to the electrodes used for training, while the black dots are used for testing. Shift inwards and onwards as shown in Figure 2. In the shift upwards, the black dots represent the training electrodes, while the white dots are the testing electrodes. In the shift downwards, the white dots signify the electrodes used for training, while the black dots signify the electrodes used for testing. Shift upwards and downwards as shown in Figure 3.

When simulating electrodes shift 10 mm and 6 electrodes damage, six randomly selected channels are replaced with Gaussian noise in both the training and testing sets. The electrode shift is same to above.

#### 2.1.3 Featured sEMG image

The HD electrodes were placed on the forearm muscle groups to record sEMG signals. The able-bodied subjects used 192 electrodes while the amputee group used 144 electrodes. For each sample, it is a 192-dimensional or 144-dimensional vector, and since we simulated electrode shift during the preprocessing stage, it becomes a 96-dimensional or 72-dimensional vector. To facilitate the extraction of spatial features, the data is transformed into a two-dimensional matrix according to the original relative placement of the collected electrodes, and then converted into an image called feature surface electromyography image (FSI). The FSI can simultaneously utilize the temporal features and the spatial features of HD-sEMG. Ninety-six or seventy-two HD electrodes were converted into matrices (for inwards/onwards shift, it is 8 × 12 or 6 × 12, and for upwards/downwards shift, it is 4 × 24 or 3 × 24). For the above matrices, the elements of the matrix were treated as the pixels of the generated FSI (Geng et al., 2016). At the same time, a linear transformation is applied to convert the original sEMG signals from (−2.5 mV, 2.5 mV) to color grayscale images (0, 255). To improve training speed and reduce internal variance shift of the data, we normalize each pixel of the FSI using the max-min. The max-min normalization is defined as:


$$x^{\prime }=\frac{x-x_{\text{min}}}{x_{\text{max}}-x_{\text{min}}}$$

Because one set of RMS values can be transformed into one FSI, and one gesture contains 30 sets of RMS values, each gesture was repeated 16 times, for a total of 9 gestures, a total of 4,320 FSIs can be generated for each subject’s data.

### 2.2 Compared models

#### 2.2.1 Locality sensitive discriminant analysis

Locality sensitive discriminant analysis (LSDA) belongs to the manifold learning algorithm (Cai et al., 2007). Its main idea is to make the similar samples in the high-dimensional data neighbors be projected closer to the low-dimensional space by maximizing the edges of different classes in each partial area, and at the same time Keep samples of different classes away from each other, so that high-dimensional data has stronger separability in low-dimensional space. This feature extraction method can achieve good results in gesture recognition.

#### 2.2.2 Convolutional neural network

CNN is a common deep learning model mainly used for processing data with grid structures (Shelhamer et al., 2017). CNN has been successful in the realm of computer vision, a technology frequently utilized in image generation, object detection, and image classification. The ability of CNN to automatically extract features from input data is its key innovation. CNN incorporates specialized layers, such as convolutional layers and pooling layers, which exploit the spatial structure of the data. CNN utilizes convolution and pooling operations to automatically extract features from data like images and performs classification or regression tasks through fully connected layers. Its hierarchical structure and parameter sharing mechanism enable outstanding performance and efficiency in handling large-scale data and complex tasks. In this experiment, we set up a total of 3 convolutional layers, with input channels of 1, 32, 64 for each layer. The kernel size is 3 × 3 and step is 1 for each layer.

#### 2.2.3 Temporal convolutional network

Temporal convolutional network (TCN) is a neural network architecture that can be used to solve time series forecasting (Bai et al., 2018). It can efficiently capture long-term dependencies in sequential data. TCN uses dilated convolutions to extend the receptive field exponentially without increasing the computational cost, enabling it to capture dependencies across long time spans. In addition, it uses causal convolution. Causal convolution ensures that each output element only depends on the preceding elements in the input sequence, making the model suitable for real-time prediction tasks. It is a one-way structure, not two-way. This means that only previous causes can affect the future. It is a strictly time-constrained model, so it is called causal convolution. In this experiment, we set the number of channels to 32, 64, 64, 32 and the kernel size to 3. The dropout is 0.3.

#### 2.2.4 Long short-term memory

Long short-term memory (LSTM) was designed to model sequential data and capture long-term dependence (Hochreiter and Schmidhuber, 1997). It can effectively transmit and retain information in long-term sequences. LSTM can also solve the problem of vanishing gradients or exploding gradients in RNN. We use it as a compared model and set the layer number to 3, hidden size to 64, and dropout to 0.2. Attention-BiLSTM is an improved model of LSTM (Zhou et al., 2016). Although LSTM can better capture long-term dependence, it can only encoder information in one direction. However, BiLSTM can process input information from both forward and backward directions. Attention is like humans who pay more attention to important information. It allows the model to dynamically assign different weights or importance to different elements of the input sequence. This enables attention to achieve better performance across a wide range of tasks. We set the same parameters as LSTM in the experiment, but with the addition of a bidirectional structure and an attention module.

#### 2.2.5 Transformer

Transformer is currently the most popular model (Vaswani et al., 2017). The key innovation of Transformer lies in its attention mechanism, which allows the model to capture global dependencies between input and output elements at different positions in the sequence. This eliminates the need for recurrent connections, making the Transformer highly parallelizable and allowing for more efficient training and inference. We set it as the compared method and set the head number to 8, encoder number to 2, and decoder number to 2. Swin-Transformer is a variant of Transformer (Liu et al., 2021), it is a new visual Transformer model suitable for computer vision tasks. It introduces a hierarchical and window-based approach to process images, enabling efficient and scalable modeling of visual information. Swin-Transformer divides the image into non-overlapping windows and operates on these windows at different scales. This hierarchical processing allows the model to capture both local and global contextual information efficiently. The advantage of the Swin-Transformer model is that it can process images of any size, has high computational efficiency and good performance.

### 2.3 Attention-deep fast convolutional neural network

#### 2.3.1 Structure of attention-DFCNN

**Figure 4:**
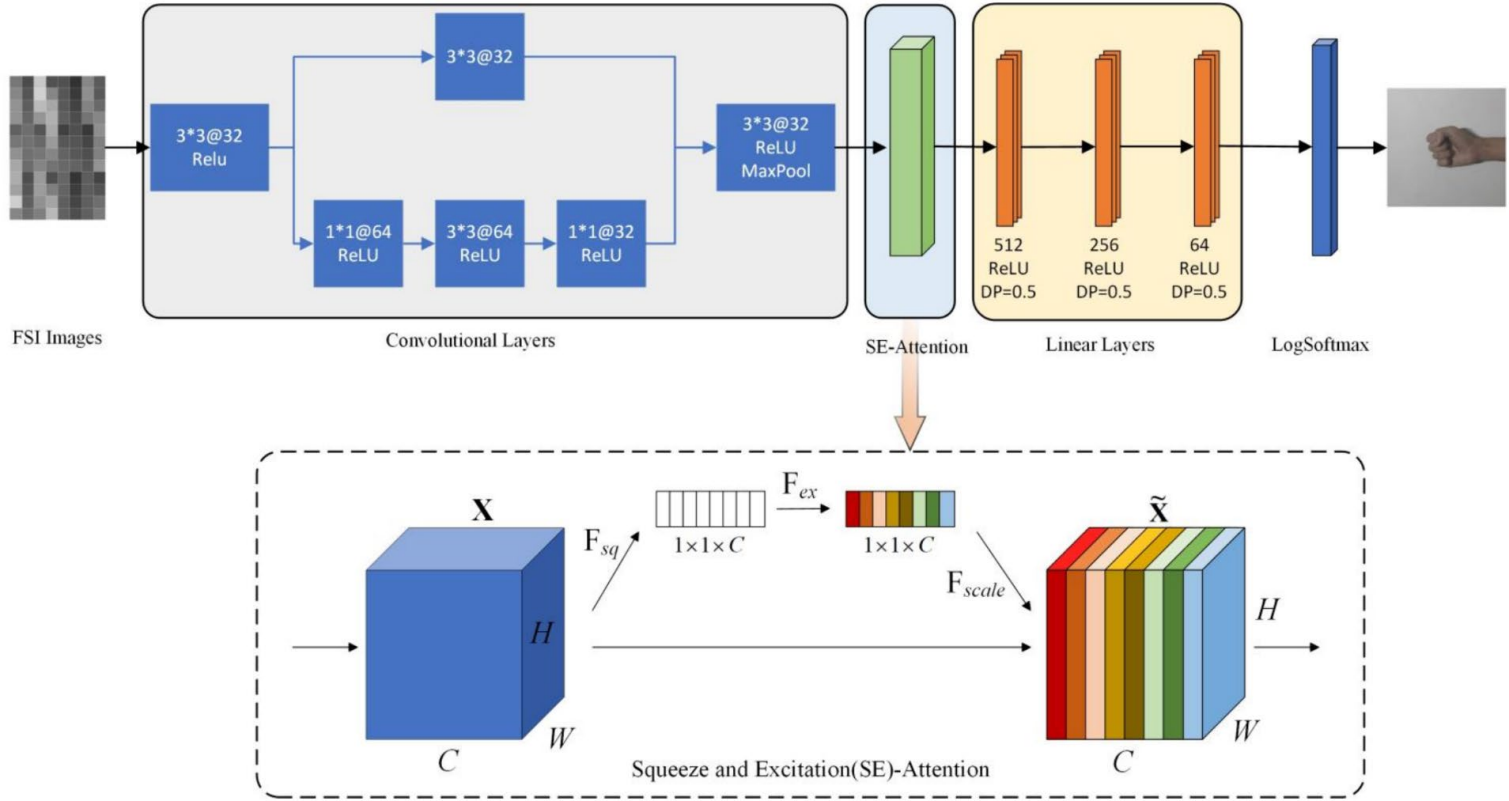
The architecture of attention-DFCNN structure. 3*3@32 represents that the kernel size of the convolutional layer is 3 × 3 and consists 32 filters. DP represents dropout. In the SE-attention structure, *H*, *W*, *C* represent the height, width and channels of the FSI, *F*_sq_ refers to the squeeze operation, *F*_ex_ refers to the excitation operation and *F*_scale_ refers to the scale operation.

We propose a new attention-DFCNN model that fully utilizes spatial and temporal features to reduce the impact of electrode shift and damage. Figure 4 depicts the architecture of the attention-DFCNN model. The small size of the kernel greatly reduces the complexity of parameter optimization.

The characteristic the attention-DFCNN model is that it uses FSI. FSI produces different signal strength distributions among different gestures. And the combination of spatial and temporal features can learn multidimensional representation information. In addition, CNN-based models can capture critical features even in the absence of some channels, effectively avoiding uneven data distributions caused by electrode shift and damage. Furthermore, by using FSI to convert 96 or 72 channels of sEMG data into 1 channel of image data, the training and inference time is accelerated, demonstrating the fast advantage of the model.

The input of the attention-DFCNN model consists of 8 × 12, 4 × 24, 6 × 12 or 3 × 24 images. The first part of the model is convolutional layers. The first convolutional layer can extract signal spatial distributions corresponding to specific muscle positions and local information in FSI using the smallest local receptive field. After the convolutional layer (3 × 3), we expand the number of channels before performing the next convolution. It can be equivalent to a local nonlinear transformer, which can map features to high dimensions, keeping the feature size unchanged, and allowing to learn more complex feature representations (Gu et al., 2018). In the convolutional layers, a residual connection is used to reduce the impact of overfitting.

After the convolutional layer is a squeeze and excitation (SE)-attention layer (Hu et al., 2018). This attention mechanism mainly consists of three parts: squeeze, excitation and scale. In the squeeze operation, a global spatial average pooling operation is applied to the feature maps and transforms the *H* × *W* × *C* feature into a 1 × 1 × *C* feature. This pooling operation aggregates information across the spatial extent of each channel, effectively summarizing the channel-level statistics. After the squeeze operation is the excitation operation. It learns to capture channel-wise relationships and generate a set of weights representing the importance of each channel. It is a multi-layer perceptron that is learnable and reduces the number of parameters through dimensionality reduction. The sigmoid function is used to obtain normalized weights between 0–1, explicitly modeling the correlation between feature channels. We designate the excitation operation as s :

$$s=F_{\mathrm{ex}}\left(z,W\right)=\sigma \left(g\left(z,W\right)\right)=\sigma \left(W_{2}\delta \left(W_{1}z\right)\right)\text{,}$$

where z represents the outputs of squeeze, δ is the ReLU function, $W_{1}$ and $W_{2}$ are the parameters of the fully connected layer, σ refers to the Sigmoid function. Finally, the scale operation scales the original features in the channel dimension by multiplying them with the width of the channel and adding them up weighted by the computed importance. The scale operation can be described as follow:

$$\tilde{X}=F_{\mathrm{scale}}\left(u,s\right)=s\;.\;u\text{,}$$

where *u* is the output before the SE-attention layer, *s* is the output of the excitation operation.

The following three hidden layers are linear layers with 512, 256 and 64 units, respectively. The last layer is a fully connected layer using the LogSoftmax function. It allows the network to learn hierarchical representations and extract high-level features from the input data. By connecting every neuron to every other neuron in the previous and next layers, it enables the network to capture complex relationships and perform sophisticated computations, and then use the LogSoftmax function to generate a probability distribution for the specific samples corresponding to multi-classes gestures.

After each convolutional and linear layer, we add ReLU nonlinear activation function. The ReLU function uses a non-linear transformation that can learn more important features and improve the generalization for different individuals and gestures. Additionally, dropouts are added to the linear layer to reduce overfitting.

#### 2.3.2 Training

In order to ensure the rationality and robustness of dividing the training sets and testing sets, we use *K*-fold cross-validation (*K* = 4). Due to converting the sEMG data into FSI during the preprocessing stage, and 4,320 FSIs were generated for each subject, so we used 3,240 FSIs for training and 1,080 FSIs for testing in each cross validation. For each subject, separate training and testing are conducted. The electrodes with shift and damage were the same for all subjects. During training, the model weights were initialized using random initialization, which can effectively break the symmetry problem in the gradients. In this experiment, eight different methods, including LSDA, CNN, TCN, LSTM, attention-BiLSTM, Transformer, Swin-Transformer, and attention-DFCNN were used on the same dataset. We built LSDA on MATLAB, and built other models on the Pytorch framework, comparing their performance on gesture recognition. All the models were trained on the same GPU (NVIDIA GeForce RTX 4090). The learning rate is 0.001 and the batch size is 128 for all the method. To speed up the convergence and save computational resources, Adam was used as the training optimizer, all the deep learning models were trained for 200 epochs to converge.

We mainly classified nine gestures and analyzed the performance differences of the eight methods. The gesture recognition accuracy of 96 electrodes for able-bodied subjects and 72 electrodes for one amputee was evaluated. To simulated the electrodes damage, Gaussian noise signals with a frequency of 50 Hz were randomly substituted for the sEMG signals collected by six electrodes in the same position in the training and test sets (Li, 2008). For 96 channels, we selected them for experimentation when the electrodes shift 10 mm or shift 10 mm and damage 6 channels. The same analysis was performed for the electrodes of amputees (72 channels) under shift and damage. The sEMG signals processed by RMS were directly used as the input of the model when using LSDA, TCN, LSTM, attention-BiLSTM and Transformer. However, for CNN, Swin-Transformer and attention-DFCNN, the sEMG signals based RMS were converted into FSI as the input.

## 3 Results

The work studied the impact of the electrode shift and damage on gesture recognition using sEMG signals. There are four directions of the electrodes shift (inwards, onwards, upwards, downwards). The classification accuracies of the attention-DFCNN model were compared with the classical method LSDA and deep learning methods including CNN, TCN, LSTM, attention-BiLSTM, Transformer and Swin-Transformer. The Wilcoxon signed-rank test was applied to evaluate the significance of our method. For the able-bodied subjects, the count of the samples is 112 (the samples contain the result of 7 subjects, the result of cross-validation (*K* = 4), and the result of shift 4 directions). For the amputee, the count of the samples is 16 (the samples contain the result of 1 amputee, the result of cross-validation (*K* = 4), and the result of shift 4 directions). The experiment showed that all the results display *p*-value <0.01. Considering the shift distance that may occur when wearing sEMG acquisition devices, our work only considered the electrodes shift of 10 mm. To verify the robustness of attention-DFCNN compared to other methods of the electrodes shift, further research was conducted by adding noise to simulate the electrodes damage during the shift.

### 3.1 The analysis of electrodes shift

The experimental results for seven able-bodied subjects with the electrodes shift 10 mm are summarized in Table 1. Four different electrode configurations (inwards, onwards, upwards, downwards) were used for the half-matrix (96 electrodes). The attention-DFCNN model achieved an average accuracy of 94.16% ± 3.91% in all configurations (inwards, onwards, upwards, downwards) and remained stable and reliable. The attention-DFCNN method outperformed other methods including CNN, TCN, LSTM, attention-BiLSTM, Transformer and Swin-Transformer. The results indicate that the proposed model can adaptively use local spatial features and is robust to the effects of electrode shift while maintaining stability across different subjects.

**Table 1:** The accuracies of 7 able-bodied subjects with the electrodes shift.

	Inwards	Onwards	Upwards	Downwards	Average
LSDA	87.79 ± 3.00	87.97 ± 3.55	93.45 ± 2.21	94.75 ± 1.92	90.99 ± 4.11
CNN	87.82 ± 4.59	89.99 ± 4.62	95.30 ± 4.04	94.88 ± 3.13	92.00 ± 5.08
TCN	82.06 ± 7.76	84.95 ± 3.69	86.88 ± 5.20	81.83 ± 10.92	83.93 ± 7.31
LSTM	82.66 ± 6.87	82.09 ± 8.86	90.59 ± 8.45	90.25 ± 4.85	86.40 ± 8.12
Attention-BiLSTM	83.65 ± 3.64	80.58 ± 9.29	88.11 ± 6.03	95.10 ± 3.15	85.19 ± 6.86
Transformer	86.91 ± 7.14	87.75 ± 9.14	93.05 ± 6.99	93.41 ± 3.03	90.28 ± 7.21
Swin-Transformer	80.72 ± 11.55	91.31 ± 4.17	95.80 ± 4.06	94.46 ± 4.33	90.82 ± 8.53
Attention-DFCNN	**90.17 ± 3.13**	**93.03 ± 3.42**	**97.17 ± 2.71**	**96.28 ± 2.02**	**94.16 ± 3.91**

Compared the attention-DFCNN method with the comparative method, all the results display *p*-value <0.01.

Table 2 shows the results for the amputee subject. As the amputee used only 144 electrode channels, 72 electrodes were used with four configurations (inwards, onwards, upwards, downwards). The overall accuracy of the proposed model (57.08% ± 2.96%) was higher than that of the other compared methods and remained stable in different configurations. The reason why the accuracy of amputee is significantly lower than that of able-bodied subjects is mainly due to a decrease in the number of electrodes used to collect sEMG signals and a decrease in the ability of the amputee to control defective muscles. At the same time, there may be errors in the task of using the phantom limb to replicate the able-limb. The proposed attention-DFCNN model is based on temporal and spatial features, which can improve robustness to electrode shift and reduce the impact on recognition accuracy.

**Table 2:** The accuracies of 1 amputee subject with the electrodes shift.

	Inwards	Onwards	Upwards	Downwards	Average
LSDA	54.15 ± 1.56	47.66 ± 2.17	50.25 ± 2.44	46.58 ± 2.10	49.66 ± 3.03
CNN	45.85 ± 2.15	49.59 ± 1.25	45.01 ± 2.45	42.95 ± 2.44	45.76 ± 2.45
TCN	40.25 ± 1.89	37.66 ± 2.05	42.01 ± 1.94	43.25 ± 2.59	41.09 ± 2.22
LSTM	41.98 ± 2.55	48.05 ± 3.58	36.98 ± 1.88	45.11 ± 2.22	43.03 ± 4.59
Attention-BiLSTM	46.12 ± 2.25	48.96 ± 3.12	41.20 ± 2.56	49.88 ± 3.89	46.54 ± 4.28
Transformer	51.02 ± 2.98	53.55 ± 1.96	46.87 ± 3.24	54.00 ± 1.95	51.36 ± 3.83
Swin-Transformer	45.55 ± 2.69	50.96 ± 2.15	46.23 ± 3.22	45.88 ± 2.55	47.16 ± 3.21
Attention-DFCNN	**59.25 ± 1.54**	**59.23 ± 2.32**	**54.00 ± 1.26**	**55.82 ± 1.48**	**57.08 ± 2.96**

Compared the attention-DFCNN method with the comparative method, all the results display *p*-value <0.01.

**Figure 5:**
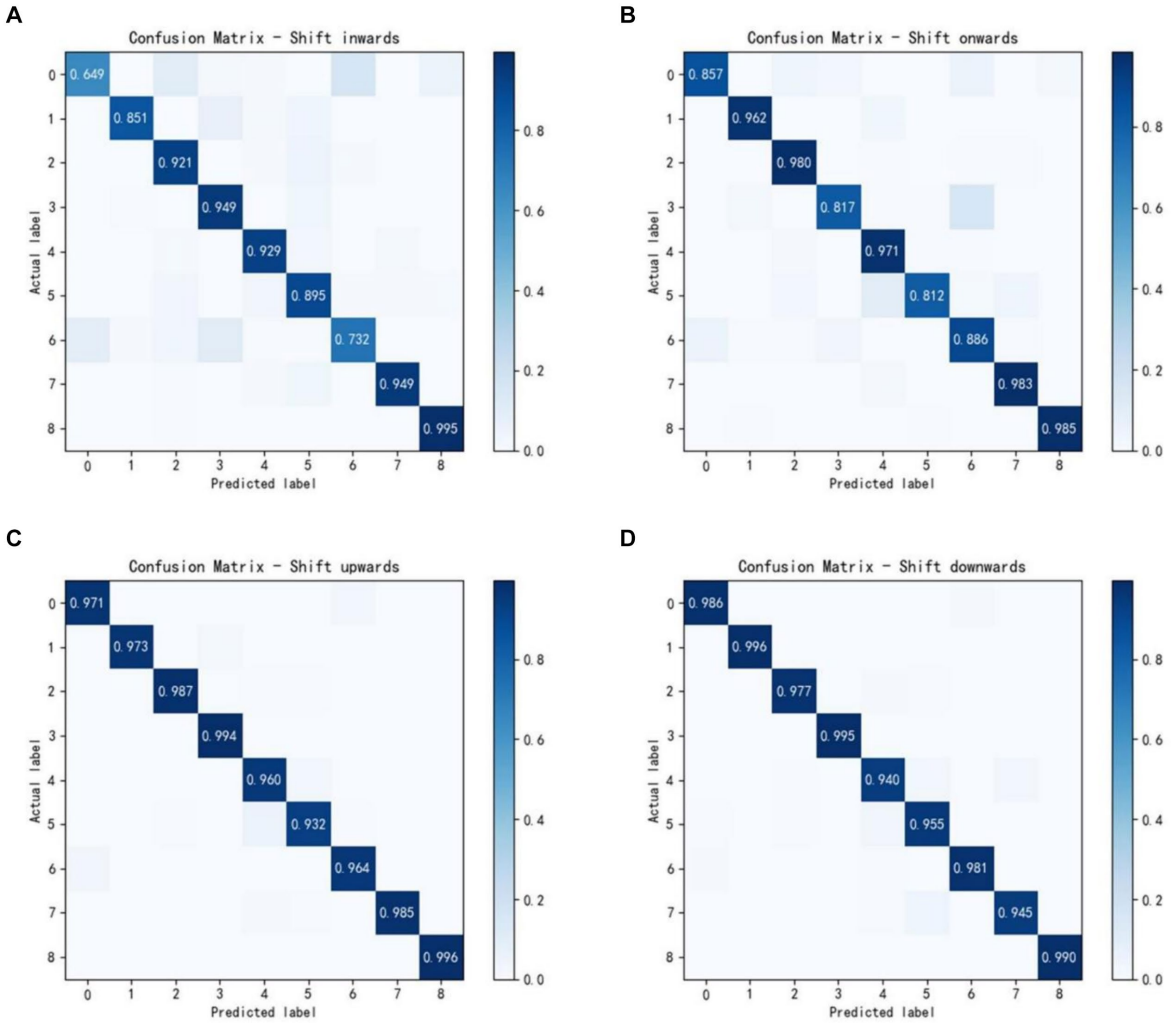
Confusion matrices of 7 able-bodied subjects with the electrodes shift 10 mm in four directions: **(A)** inwards; **(B)** onwards; **(C)** upwards; **(D)** downwards.

Figure 5 shows the confusion matrices of the average accuracies of 7 able-bodied subjects using the attention-DFCNN model. The four confusion matrices represent the electrodes shift 10 mm in four directions (inwards, onwards, upwards and downwards), and the coordinate axes 0–8 of the matrices represent nine different gestures. In most gesture recognition, the attention-DFCNN model can achieve more than 90% accuracy.

### 3.2 The analysis of electrodes shift and damage

The performance of the attention-DFCNN method was analyzed with the impact of electrodes shift and noise channels. When the electrodes shift 10 mm, the half matrices (96 electrodes) were used and added noise to the random 6 channels of the test and training datasets. The results are shown in Table 3. For seven able-bodied subjects, the recognition accuracy of attention-DFCNN still showed higher accuracy than other methods, with all the methods displaying *p*-value <0.01. Even with 10 mm electrode shift and noise channels, the proposed model can detect the noised channels while training and apply slight weight to them in order to lessen the effect caused by noises.

**Table 3:** The accuracies of 7 able-bodied subjects with the electrodes shift and damage.

	Inwards	Onwards	Upwards	Downwards	Average
LSDA	80.46 ± 5.43	79.40 ± 4.62	87.11 ± 3.46	89.60 ± 3.46	84.14 ± 6.00
CNN	81.66 ± 3.11	85.01 ± 3.72	91.36 ± 5.24	82.62 ± 4.66	85.16 ± 5.57
TCN	78.52 ± 10.79	80.14 ± 7.64	85.80 ± 7.41	82.79 ± 7.37	81.81 ± 8.43
LSTM	75.09 ± 6.09	83.45 ± 8.72	78.94 ± 6.65	76.33 ± 10.16	78.45 ± 8.28
Attention-BiLSTM	74.31 ± 7.15	83.80 ± 8.36	85.30 ± 6.84	81.03 ± 5.63	81.11 ± 7.92
Transformer	78.01 ± 7.77	87.87 ± 7.19	83.08 ± 7.17	79.91 ± 7.13	82.04 ± 7.75
Swin-Transformer	70.19 ± 9.45	89.22 ± 3.28	85.78 ± 9.01	85.62 ± 7.36	82.70 ± 10.42
Attention-DFCNN	**86.74 ± 3.84**	**90.13 ± 5.52**	**91.76 ± 5.06**	**92.83 ± 4.76**	**90.37 ± 5.13**

Compared the attention-DFCNN method with the comparative method, all the results display *p*-value <0.01.

**Figure 6:**
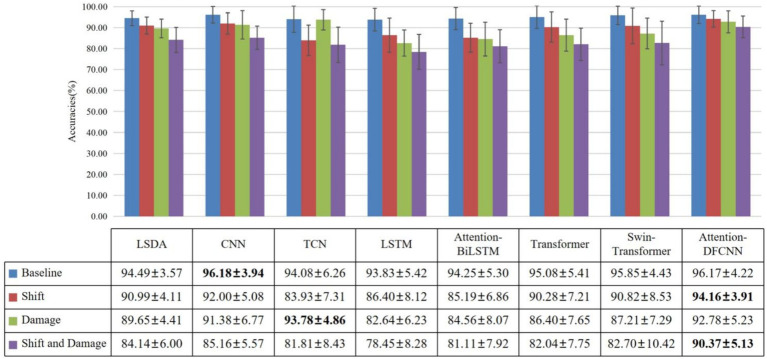
The accuracies of 7 able-bodied subjects with base, shift 10 mm, damage 6 channels, shift and damage. Base represents no electrode shift and damage, shift represents only shift 10 mm, damage represents only damage 6 channels, shift and damage represent shift 10 mm and damage 6 channels.

We also compared the results of shift and damage with the baseline test, only shift test, and only electrode damage test (as shown in Figure 6). We can observe that the attention-DFCNN model has the smallest decrease in accuracy. The results of shift and damage showed a 5.8% decrease in accuracy compared to the baseline test, and a 3.79% decrease compared to only shift test. At the same time, it can be found that the attention mechanism and convolutional structure can reduce the impact of electrodes shift on accuracy, and the dilated convolution of TCN can almost avoid the impact of electrode damage. In addition, by comparing the experimental results, it can be found that the larger standard deviation values are caused by some models not being able to adapt to electrode shift. Because the standard deviation values of all models were low in the baseline experiment. Due to insufficient training data, it is difficult to learn enough features for gesture recognition in complex situations such as electrode shift. In some cases, overfitting has occurred, so the recognition accuracy varies greatly for different subjects.

The same analysis was used in one amputee subject as shown in Table 4, seven methods were used to analyzed the performance. The attention-DFCNN model achieved a recognition accuracy of 54.27% ± 5.45% (*p* < 0.01) when the electrodes shift 10 mm and electrode damage, and the difference between diverse transfer directions was not significant.

**Table 4:** The accuracies of 1 amputee subject with the electrodes shift and damage.

	Inwards	Onwards	Upwards	Downwards	Average
LSDA	**51.02 ± 2.05**	42.85 ± 1.23	34.21 ± 1.56	34.88 ± 2.52	40.74 ± 6.84
CNN	42.00 ± 1.44	45.24 ± 1.99	37.51 ± 2.20	33.95 ± 3.02	39.68 ± 4.30
TCN	40.07 ± 2.12	34.54 ± 1.96	43.06 ± 2.11	34.26 ± 3.25	38.40 ± 3.38
LSTM	42.12 ± 2.55	36.54 ± 2.54	33.95 ± 4.23	33.89 ± 2.87	36.63 ± 3.85
Attention-BiLSTM	40.52 ± 2.12	42.78 ± 1.60	40.96 ± 3.25	36.10 ± 2.56	40.09 ± 3.54
Transformer	41.32 ± 3.12	55.46 ± 2.95	40.32 ± 3.66	58.44 ± 4.01	48.89 ± 9.32
Swin-Transformer	45.32 ± 3.24	52.14 ± 2.86	38.42 ± 2.65	49.41 ± 3.84	46.32 ± 6.95
Attention-DFCNN	45.82 ± 1.87	**56.43 ± 1.32**	**54.32 ± 2.13**	**60.51 ± 1.54**	**54.27 ± 5.45**

Compared the attention-DFCNN method with the comparative method, all the results display *p*-value <0.01.

## 4 Discussion

We proposed the attention-DFCNN model, which is a fast and efficient framework based on FSI to mitigate the effects of electrode shift and damage. We validate that the model improves the performance under two situations: shift 10 mm and shift 10 mm with damage 6 channels. Attention-DFCNN achieved accuracies of 94.16 and 90.37% for able-bodied subjects. We also improved the recognition accuracy of the amputee, reaching as high as 57.08 and 54.27. While some other methods such as TCN and LSTM experience a significant drop in accuracy when electrode shift occurs, attention-DFCNN maintains a high recognition accuracy with minimal variations across different shift types. The attention-DFCNN model, based on spatial and temporal features, also exhibits robustness to electrode damage, which has only a slight impact on all participants. The deep learning model is able to assign different weights to signals corresponding to electrodes. As a result, the attention-DFCNN model can automatically detect noisy or missing signals from an alternative perspective, thus exhibiting better stability and robustness to electrode shift and damage.

Compared to research using the same dataset (Stango et al., 2015), our model has an advantage in recognition accuracy with the impact of electrodes shift when using data from able-bodied subjects, especially inwards shift and onwards shift. This is because the deep learning approach utilizes the spatial features of the data and can selectively assign weights to the signal. However, for the amputee, our recognition accuracy significantly decreases. This may be because we use the deep learning model to achieve gesture recognition, and the data preprocessing method is different from that of Stango et al. (2015), and our training data is too limited, which makes it difficult for the model to learn enough features, which is unfavorable for deep learning models. Compared with other studies, for example, in this study (Ameri et al., 2020), they used transfer learning to reduce the impact of electrode shift on sEMG recognition. This method may have better training and inference time than our model, but it requires recalibration during use. Moreover, when there is electrode damage, the accuracy of recognition may decrease significantly, and our model, due to the use of high-density electrodes, will not produce this situation. In addition, compared with the study by He et al. (2020), they proposed a novel approach to avoid the influence of electrode shift by directly adjusting the worn electromyography collection device. The corrected recognition accuracy is almost the same as that without electrode shift. Our model has no advantage in recognition accuracy compared to their method, but in reality, it may not be convenient to adjust the wearing position multiple times to ensure recognition accuracy when worn for a long time. And our method can deal with the occurrence of electrode damage. We believe that our current algorithm for controlling prosthetics for amputees is still difficult, but it can be used for able-bodied individuals to remotely control robotic arms to perform specific tasks in hazardous areas (Park et al., 2019), or assist people in muscle strength training (Meattini et al., 2020).

## 5 Conclusion

We introduced an attention-DFCNN model based on spatial and temporal featured sEMG image to solve the problem of electrode shift and damage in gesture recognition when this situation occurs. At present, although able-bodied subjects have achieved excellent performance, the recognition accuracy of amputees is relatively low. In the future, we plan to utilize the idea of transfer learning to enhance the recognition performance of amputees by utilizing data from able-bodied subjects.

## Data availability statement

The original contributions presented in the study are included in the article/supplementary material, further inquiries can be directed to the corresponding author.

## Author contributions

CL: Conceptualization, Investigation, Software, Supervision, Writing – original draft, Writing – review & editing. YW: Data curation, Formal analysis, Methodology, Validation, Visualization, Writing – original draft, Writing – review & editing. MD: Formal analysis, Resources, Validation, Visualization, Writing – review & editing.
